# The peatlands developing history in the Sanjiang Plain, NE China, and its response to East Asian monsoon variation

**DOI:** 10.1038/srep11316

**Published:** 2015-06-16

**Authors:** Zhenqing Zhang, Wei Xing, Guoping Wang, Shouzheng Tong, Xianguo Lv, Jimin Sun

**Affiliations:** 1 Key Laboratory of Wetland Ecology and Environment, Northeast Institute of Geography and Agroecology, Chinese Academy of Sciences, China; 2 key Laboratory of Cenozoic Geology and Environment, Institute of Geology and Geophysics, Chinese Academy of Sciences, China

## Abstract

Studying the peatlands accumulation and carbon (C) storage in monsoonal areas could provide useful insights into the response of C dynamics to climate variation in the geological past. Here, we integrated 40 well-dated peat/lake sediment cores to reveal the peatlands evolution history in the Sanjiang Plain and examine its links to East Asian monsoon variations during the Holocene. The results show that 80% peatlands in the Sanjiang Plain initiated after 4.7 ka (1 ka = 1000 cal yr BP), with the largest initiating frequency around 4.5 ka. The mean C accumulation rate of peatlands in the Sanjiang Plain exhibits a synchronous increase with the peatlands expansion during the Holocene. Such a peatlands expanding and C accumulating pattern corresponds well to the remarkable drying event subsequent to the Holocene monsoon maximum. We suggest that in addition to the locally topographic conditions, Holocene variations of East Asian summer monsoon (especially its associated precipitation) have played a critical role in driving the peatlands initiation and expansion in the Sanjiang Plain.

Peatlands as one of the largest biosphere carbon (C) reservoirs and CH_4_ sources have played an important role in global carbon cycle and climate changes during geological past^1^,^2^,^3^. Understanding the responses of these C-rich ecosystems to past climate changes could provide useful insights into projecting the fate of peatlands C in the future^4^,^5^,^6^. During last decades, numerous works have been done to reveal the peatlands dynamics to the local climate changes. It has been well documented that peat accumulates whenever the rate of organic matter production exceeds the rate of decay, and which is mainly controlled by the local temperature and moisture conditions^7^. Generally, a warmer condition in growing seasons will favor more primary production and in turn a higher peat accumulation rate in peatlands although it may cause more peat decomposition, and a much colder climate in winter will be more favorable to preserve more peat from being oxidized and decomposited^8^,^9^. In this sense, a higher degree of climatic seasonality generally leads to a higher peat accumulation rate^5^. Such a contention is supported by a mid-high latitude distributing pattern of the northern peatlands, where the climate is characterized by the remarkable seasonality^10^.

In addition to temperatures, the local moisture conditions can also generate a significant influence on peat accumulation. Modeling experiments in wetlands show that a wetter condition is roughly more productive to peat accumulation with higher primary production but lower peat decomposition^11^. While a few works have tentatively revealed the response of peat accumulation to past moisture conditions, the results show much different controls of the moisture conditions for peatlands expansion and C accumulation^10^,^12^,^13^. For example, the peat deposits in Alaska accumulate more quickly in much drier conditions^19^, and a considerable number of world peatlands initiated during the Last Glacial Maximum, a relatively cold and dry interval^10^,^14^. Such an inverse correlation between the peat accumulation and moisture conditions seems to be inconsistent with the modeling results. So, the mechanisms of peatlands response to the moisture conditions may be more complicated than anticipated, and clarification of this issue will require high-quality records from more climatically sensitive locations.

The Sanjiang Plain known as the largest fresh-water wetland area is located in the northern monsoon marginal region, making it a particularly sensitive region to East Asian monsoon variations^15^. In this paper, we integrated 40 well-dated peat cores to reveal peatlands initiation and C accumulation histories in the Sanjiang Plain, and discuss their relations to the East Asian monsoon circulations during the Holocene.

## Results

### Sampling and material

**Figure 1:**
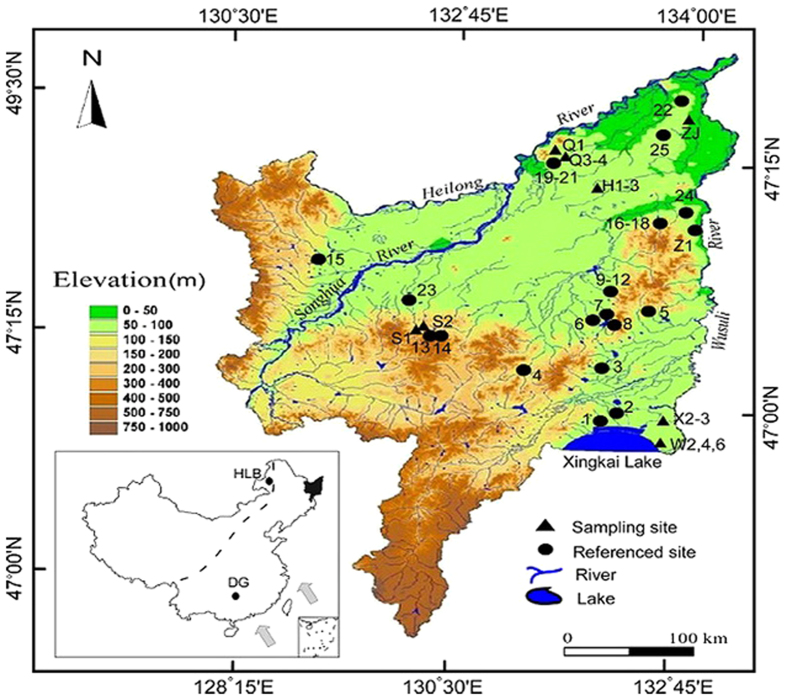
Digital elevation model of the Sanjiang Plain, which was generated by Zhenqing Zhang using ArcGIS 10.0. The solid triangles and circles in black color indicate the sampling sites and the sites of peat cores with based ages mentioned in the text respectively. See Table 2 for sites information and references. In inset figure, the current northern limit (dashed line) of the East Asian Summer monsoon with its direction indicated by the arrows, the locations of the Sanjiang Plain (highlighted in black area), the HLB and DG profiles (solid circles) mentioned in the text are shown.

**Figure 2:**
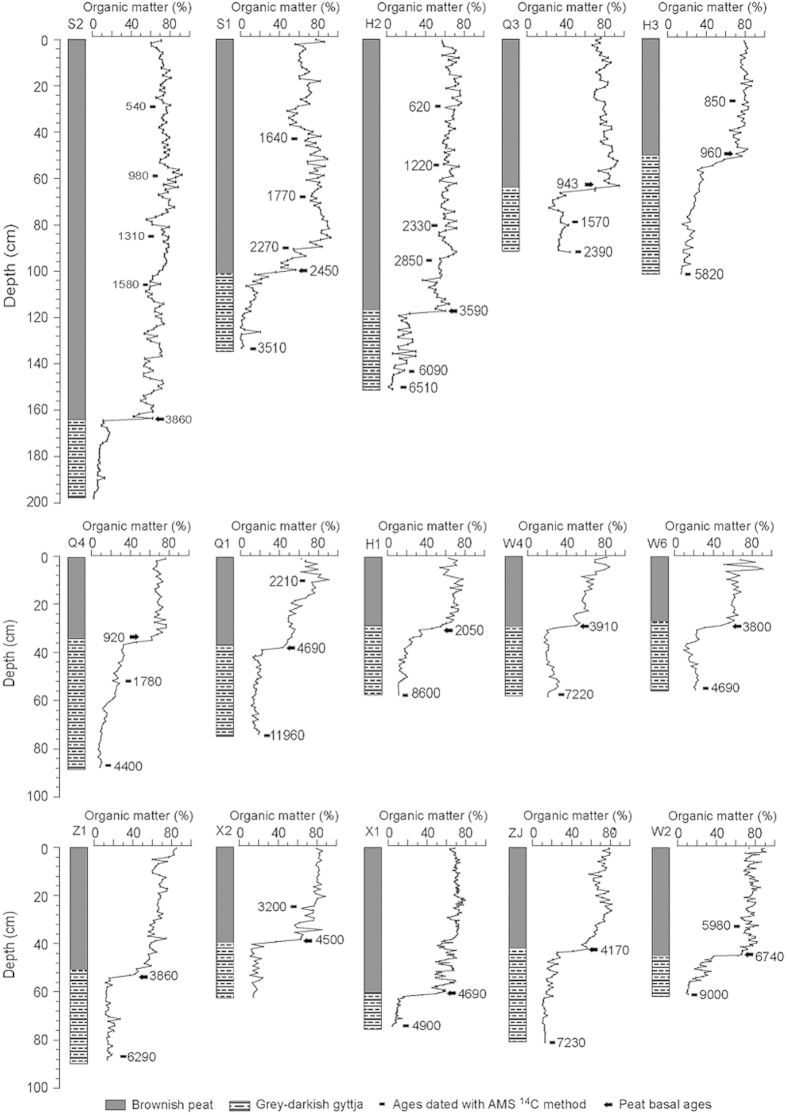
Stratigraphy and organic matter content of 15 peat/mud cores from the Sanjiang Plain. The calibrated AMS ages are marked beside the organic matter curves with the solid rectangles indicating the depth of the dating samples. The solid arrow was used to indicate the basal age of peat accumulations for each core.

During May to September in 2012, a thorough investigation was performed in the Sangjiang Plain to ascertain the modern peatlands distribution, and 15 well-preserved peatlands were found and well studied in this paper. In the central region of each peatland, a deposit core was collected using a Russia peat corer and 15 peat/mud cores in total were gained (Fig. 1). According to lithologic properties, all these cores can be subdivided into two parts (Fig. 2): the typical brownish peat layers above and the grey-blackish mud deposits below. All samples were collected with 1-cm-thick interval from each core for laboratory analysis.

### Lithology and chronology

**Figure 3:**
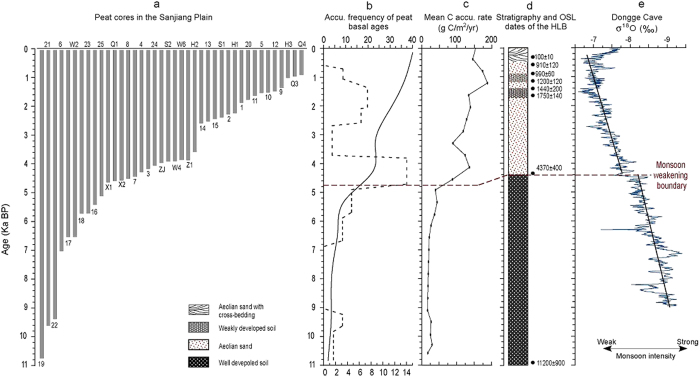
Schematic figures indicating the grouping method used to calculate the frequency (dash line in **b**) and accumulating frequency (solid line in **b**) of basal ages with assembling 40 peatlands initiation chronologies (**a**) in the Sanjiang Plain. Mean C accumulation rate calculated from LOI results of 15 peatland cores in the Sanjiang Plain (**c**). The East Asian summer monsoon variations indicated by the soil-sand sequence of HLB in the Hulun Buir Desert (**d**) and the stalagmite δ^18^O variations in Dongge Cave. The dashed line in figure was used to mark the remarkable monsoon weakening event at mid Holocene.

We used 40 basal peat ^14^C ages including 15 dates in the present study (Fig. 2 and Table. 1 ) and 25 dates from published sources (Table. 2) across the Sanjiang Plain (site locations in Fig. 1) to assess the temporal and spatial pattern of peatlands initiation. According to visual inspection and organic matter contents variation, most of the 15 collected peat cores can be subdivided into two parts: the lacustrine mud deposits with a lower organic content of ~20% in the lower part, and the overlying typical peat deposits with much higher organic contents of >50% (Fig. 2). The AMS dating results indicate that although the peatlands occurrences in the Sanjiang Plain cover a wide range of the Holocene, 80% of them concentrate in the last 4.7 ka (Fig. 3a,b) and the largest initiating frequency occurs around 4.5 ka. Both the curves of the accumulating frequency of peat basal ages and mean C accumulation rate in the Sanjiang Plain exhibit a similar variation trend, as both of them show relatively low and stable values before 4.7 ka and gradually increasing trends thereafter (Fig. 3b,c).

## Discussion

Generally, the peatlands initiation is marked by the appearance of peat layers in the geological past, and the peat deposits are defined by a high ratio of the organic matter contents. While such a definition varies largely among different countries with the organic matter contents changing from 40% to 70%^24^. Here, a median value of 50% was employed as an indicator of the peatlands initiation. With the peat basal ages of 40 peat cores and high-resolution C contents of 15 cores, we tried to the peat initiation and C accumulation history of peatlands in the Sanjiang Plain.

As shown in Fig. 3b,c, both the accumulating frequency of peatlands initiation and the mean C accumulation rate exhibit much similar variations, implying the casual relations between the two records in the Sanjiang Plain. For the interval before 4.7 ka, only a few peatlands (~20% of the total peatlands) occurred in certain locations in the Sanjiang Plain, when most depressions in the plain were dominated by shallow lakes, which is indicated by the lacustrine mud deposits with relative low organic matter contents of ~20%. Comparing the peat layers, such widespread lacustrine deposits with lower organic matter contents and accumulation rates can only generate a low and stable mean C accumulation rate before 4.7 ka. Thereafter, most of the peatlands (~80% of the total peatlands) occurred, leading to the increase of mean C accumulation rate. The interval is highlighted by a rapid peatlands expansion stages with the highest peatlands initiation frequency and the much higher rate of the mean C accumulation spanning 4.7–3.8 ka.

The present climate in the plain belongs to the temperate humid or sub-humid continental monsoon climate with relative higher mean annual precipitations^25^. In addition to the warm and wet climate, such a low-relief area with low slope grade is favorable for the development of wetlands^24^. A recent survey shows that over 70% of the plain has been dominated by fresh-water wetlands, and thus it is known as the largest fresh-water wetlands area in China^26^. While in the geological history, the lake-wetland which is so-called terrestrialization process as one of the three main peatland process with paludification, often depends on both allogenic (climate) and autogenic (ecolological) processes. And in the Sanjiang Plain, such a transition was a quick process considering the sharp boundary between the lacustine mud and peat sections. While the autogenic process (e.g. ecological evolution) is commonly accepted as much slow course of more than hundreds or thousands of years, thus it can hardly serve a dominant role in driving the rapid peatlands initiations within several decades.

Considering the prevalent monsoon climate in the Sanjiang plain, the peatlands occurrences and C accumulation pattern may be potentially linked with the monsoon variations during the Holocene. In the recent decades, numerous works have been done to reveal the monsoon evolution on different time scales^15^,^27^,^28^,^29^,^30^,^31^,^32^, and most of the records indicate a much warmer and wetter interval during the early or early-mid Holocene, corresponding to the Holocene monsoon maximum^27^,^28^,^29^,^30^,^31^,^32^. In low-mid latitudes of China, stalagmite δ^18^O has been widely employed as a climate-sensitive proxy for monsoon variation, as its values usually become lower when the Asian summer monsoon intensifies, and vice versa^27^. Such an anticorrelation is also observed in modern precipitation records near the cave site^33^. In northeastern China, the alternations of sand accumulation and paleosol development in desert regions are regarded as the direct indicators for the monsoon variations in the geological past^28^,^32^. As the soil development requires a much wetter/warmer climate and better vegetation cover comparing with the drier climate during the aeolian sand accumulation, in this context, the alternations of aeolian sand and paleosols are mainly controlled by the changes of summer monsoon strength. Here, we combined two high-resolution and absolutely-dated monsoon records from the Dongge Cave (DG)^27^ in southern China and the Hulun Buir Desert (HLB)^28^ in northeastern China respectively (site locations in Fig. 1), to discuss and reveal the relationships between peatlands development and monsoon variation in the Sanjiang Plain.

**Figure 4:**
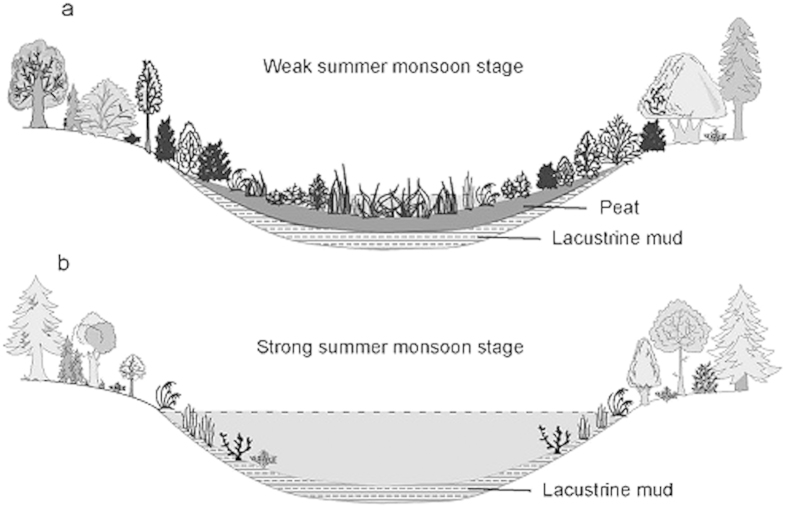
Schematic figures indicating the decline of East Asian summer monsoon plays a driving role in lake-peatland transition during Holocene. They were drawn by Zhenqing Zhang using Canvas 15.0.

During the interval before 4.7 ka, the widespread shallow lakes in the Sanjiang Plain indicate a much wetter environment, and in turn a strong summer monsoon interval considering the prevalent monsoon climate in the study regions (Fig. 4b). The interval corresponds well with the well-developed soil sections in the HLB before 4.4 ka in spite of a 300 yr discrepancy, which is acceptable in view of the 400 yr error of the OSL dating at 4.4 ka^28^, and relatively lower values of δ^18^O in the DG^27^(Fig. 3). Furthermore, such a strong monsoon interval during the early and mid Holocene has been widely documented in lake sediments^31^, eolian deposits^32^, accretionary soils^15^ and peat accumulations^34^ in monsoonal regions. While with the gradual decline of the summer monsoon strength and its associated precipitation during the mid-late Holocene^27^,^34^, the paleolakes in the Sanjiang Plain began to dry out and a number of peatlands initiated around 4.5 ka (Fig. 4a). Additionally, it is worthy to stress that the lacustrine mud layers were vitally important for the subsequent peatlands initiation, as they provided a nutrient-rich base for peatlands vegetation growing, and also a water-retaining layer for the subsequent peatlands developing. This might explain why few peatlands developed before 11.0 ka with the relative weak summer monsoon. As the weak monsoon before 11.0 ka would limit number of lakes on the landscape, and this is entirely different situation comparing to the change from abundant lakes during maximum monsoon intensity in the early Holocene to lake dry-up and conversion to wetlands in a dry mid-Holocene.

It worth noting that although 80% of the wetlands in Sangjiang Plain initiated after the remarkable monsoon decline at 4.7 ka, their initiations were not limited to that age but covered a wide range of 4.7–0.9 ka. Here, we suggest that the age discrepancies of the peatlands initiations should be attributed to the local site-specific conditions of topography, such as basin/lake depths and sizes. As deeper lakes/basins certainly take longer to respond to the same magnitude/speed of climate change than shallow lakes. While nowadays, the depths or sizes of the studied basins in geological past are hard to ascertain considering the natural landforms in the Sanjiang Plain have been seriously destroyed by human activities. In spite of this, we still accept the fact that there must be some discrepancies among the topographies of different basins, which should partly account for the responding discrepancies of the peatlands initiation to late-Holocene monsoon variations. Moreover, even during the late Holocene with the relative weak monsoon strength, there is still a more rapidly monsoon weakening trend comparing the previous stage. Thus, in addition to local topographic conditions, the gradual declination of the summer monsoon would further strengthen the discrepancies of the peatlands initiations in the Sanjiang Plain during the late Holocene.

## Methods

### Regional setting

**Figure 5:**
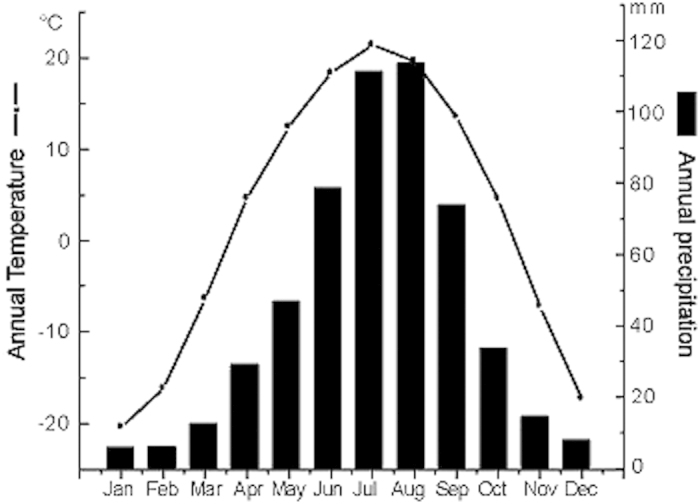
Climate diagrams showing monthly temperature and precipitation in the Sanjiang Plain. All data were from climate normal for the period 1957–2000 at meteorological stations in the Sanjiang Plain.

The Sanjiang plain (129°11′–135°05′E, 43°49′–48°27′N) located in NE China (Fig. 1) is a huge alluvial plain crossed by three major rivers: Heilong River, Wusuli River and Songhua River. It has a total area of 10.9 × 10^6^ ha, an altitude of <200 m and a slope grade of <1:10,000. The present climate of the plain belongs to the temperate humid or sub-humid continental monsoon climate. The mean annual temperature ranges from 1.4 to 4.3 °C, with average maximum of 22 °C in July and average minimum −18 °C in January. The mean annual precipitation is 500–650 mm and 80% of rainfall occurs between May and September^35^(Fig. 5).

In addition to the warm and wet climate, such an area of low-relief is favorable for the development of wetlands. A recent survey shows that over 70% of the plain has been dominated by fresh water wetlands developing in ancient riverbeds and waterlogged depressions^25^. Peatlands with a total area of 3.3 × 10^4^ ha have developed in certain topographic conditions during Holocene or earlier^24^.

### Laboratory analysis

Subsamples with a volume of 3 cm^3^ were prepared for loss-on-ignition (LOI) with sequential combustion at 500 °C and 900 °C to estimate organic matter and carbonate contents respectively^36^. Bulk density with 1 cm interval of each peat core was calculated with the dry weight and volume of each subsample. Ash-free (organic matter) bulk density was calculated from the measurements of bulk density and organic matter contents. Apparent carbon accumulation rates were calculated using calibrated AMS ^14^C ages, ash-free bulk density measurements and C contents of peat organic matter in peatlands (using 52% C in peat organic matter^37^). The mean C accumulation rate (Fig. 4c) was calculated for each 400-year bin using time-weighted averaged C accumulation rates of 15 cores showed in Fig. 2.

Base on visual inspection and LOI analysis, only the samples with a dominant component of plant residues and organic matter contents >50% were regarded as the peat deposits. While the grey-blackish mud with organic matter contents <30% was regarded as lacustrine deposits (Fig. 2). Most of the subsamples for AMS dating were collected according to lithological changes, and they were all dated with an accelerator mass spectrometry system at the Institute of Earth Environment, CAS. The AMS ^14^C dates were calibrated into calendar ages using the program Calib 7.02 based on the INTCAL 13 calibration dataset^38^ (Table. 1).

### Data analysis

To calculate the frequency of peatlands initiation, all the ages were grouped roughly into 500-year bins with additional considerations as follows: if a date in bin A has a discrepancy of no more than 100 yr with another date in bin B, we grouped the two dates in bin A (If A < B), otherwise we grouped the two dates in two different bins if the discrepancy >100 yr, indicating the two peatlands initiation stage (Fig. 3). Such an improved grouping method could avoid grouping the two neighboring dates into much different peat expansion stages, as they are more likely within the same stage considering several decades dating error of the each date. Accordingly, an accumulating frequency carve can be drawn based on the frequency of the 40 peat basal ages from the Sanjiang Plain.

## Additional Information

**How to cite this article**: Zhang, Z. *et al.* The peatlands developing history in the Sanjiang Plain, NE China, and its response to East Asian monsoon variation. *Sci. Rep.* **5**, 11316; doi: 10.1038/srep11316 (2015).

## Figures and Tables

**Table 1:** **AMS radiocarbon dates of samples from16 peat/mud cores in the Sanjiang Plain.**

**Site#**	**Lab number**	**Depth (cm)**	**Dating material**	**δ^13^C (‰)**	**AMS ^14^C age (^14^C yr BP)**	**Calibrated ^14^C age (2σ) (cal yr BP)**
S2	XA7553	27	Plant residues	−37.78	550 ± 24	520–560
S2	XA7592	60	Plant residues Organic matter	−38.64	1088 ± 24	940–1010
S2	XA7542	84	Plant residues	−30.36	1381 ± 26	1280–1340
S2	XA7543	107	Plant residues Organic matter	−24.56	1673 ± 32	1520–1630
S2	XA7570	165	Plant residues	−30.14	3542 ± 26	3810–3900
S1	XA7561	44	Plant residues	−34.77	1733 ± 24	1590–1700
S1	XA7562	67	Plant residues Organic matter	−30.51	1847 ± 23	1710–1830
S1	XA7540	92	Plant residues	−30.14	2173 ± 25	2220–2310
S1	XA7563	100	Plant residues Organic matter	−30.52	2446 ± 28	2360–25402
S1	XA7541	135	Organic matter	−29.80	3268 ± 27	3450–3570
H2	XA7572	29	Plant residues Organic matter	−32.12	606 ± 23	580–650
H2	XA7573	60	Organic matter	−30.91	1243 ± 24	1170–1270
H2	XA7574	80	Plant residues Organic matter	−31.54	2295 ± 25	2310–2350
H2	XA7576	95	Plant residues Organic matter	−29.89	2759 ± 28	2780–2930
H2	XA7577	116	Plant residues	−30.40	3335 ± 25	3550–3640
H2	XA7578	140	Plant residues Organic matter	−31.76	5313 ± 33	5990–6190
H2	XA7579	148	Organic matter	−29.89	5736 ± 28	6450–6570
Q3	XA7583	65	Plant residues	−33.75	1021 ± 24	920–970
Q3	XA7584	80	Plant residues Organic matter	−36.03	1660 ± 24	1530–1620
Q3	XA7593	92	Organic matter	−29.69	2188 ± 25	2330–2440
H3	XA7580	26	Plant residues Organic matter	−30.72	922 ± 25	790–920
H3	XA7581	50	Plant residues	−30.86	1055 ± 23	930–990
H3	XA7582	100	Organic matter Organic matter	−30.16	5061 ± 28	5740–5900
Q4	XA7587	57	Plant residues	−26.67	1852 ± 25	1720–1840
Q4	XA7588	83	Organic matter	−29.51	3950 ± 26	4350–4450
Q1	XA7589	11	Plant residues Organic matter	−26.36	2244 ± 25	2160–2270
Q1	XA7537	38	Plant residues	−27.43	4165 ± 28	4610–4770
Q1	XA7590	69	Organic matter	−27.77	10223 ± 35	11820–12090
H1	XA7567	30	Plant residues Organic matter	−29.16	2066 ± 23	1990–2120
H1	XA7568	60	Organic matter	−27.39	7830 ± 32	8540–8660
W4	XA7534	36	Organic matter	−24.57	3606 ± 28	3840–3980
W4	XA7533	60	Organic matter	−26.34	6277 ± 36	7160–7280
W6	XA7538	30	Plant residues	−23.14	3528 ± 25	3720–3880
W6	XA7537	53	Organic matter	−27.43	4165 ± 28	4610–4770
Z1	XA7539	52	Organic matter	−26.01	3547 ± 26	3810–3910
Z1	XA7528	90	Organic matter	−21.73	5476 ± 29	6260–6310
X2	XA7564	24	Plant residues Organic matter	−29.49	3005 ± 25	3140–3250
X2	XA7560	40	Organic matter	−29.38	3983 ± 27	4460–4520
X1	XA7558	60	Plant residues Organic matter	−29.99	4194 ± 39	4610–4770
X1	XA7559	76	Organic matter	−30.58	4278 ± 28	4830–4770
ZJ	XA7569	44	Organic matter Organic matter	−29.45	3797 ± 26	4090–4250
ZJ	XA7591	80	Organic matter	−32.75	6308 ± 31	7170–7290
W2	XA7529	32	Organic matter	−22.21	5258 ± 27	5930–6030
W2	XA7530	45	Organic matter	−22.01	5937 ± 33	6670–6810
W2	XA7527	62	Organic matter	−22.01	8062 ± 33	8970–9030

**Table 2:** **Radiocarbon dates and location of each site mentioned in this paper.**

**Site No.**	**Site Names**	**Latitude(N)**	**Longitude (E)**	**Depth of peat (cm)**	**^14^C date**	**error**	**Basal age (Cal yr BP)**	**References**
1	Xingkaihu	45°19′	132°9′	140	1486	140	1733	16
2	Yangmu	45°36′	132°25′	145	3400	342	2325	17
3	Huling	45°49′	132°56′	70–80	3775	80	4188	18
4	Xinshugongshe	45°55′	130°34′	55–60	3991	82	4443	18
5	Shuguang	46°10′	133°03′	320–330	1600	70	1487	18
6	Dongsheng	46°29′	132°28′	140–150	6955	105	6955	18
7	Dongsheng	46°37′	132°31′	N/A	4417	307	4417	19
8	Jinlong	46°32′	132°35′	136	4027	308	4503	20
9	Qinghe1	46°35′	132°58′	85–90	1425	90	1350	18
10	Qinghe 2	46°35′	132°58′	N/A	1585	90	1470	18
11	Baoqing	46°36′	132°57′	120	1585	90	1585	17
12	Qinghe 3	46°36′	132°57′	N/A	1610	200	1560	18
13	Shenjiadian1	46°36′	130°38′	N/A	2470	80	2470	21
14	Shenjiadian2	46°36′	130°38′	195–200	2540	80	2541	17
15	Huachuan	46°37′	132°31′	N/A	4417	307	2388	17
16	Bielahonghe1	47°01′	130°43′	195	2375	167	5347	22
17	Bielahonghe2	47°31′	134°04′	168	4615	75	6465	22
18	Bielahonghe3	47°31′	134°04′	N/A	5650	95	5650	17
19	Qindeli1	47°55′	133°13′	196	9420	70	10651	22
20	Qindeli2	47°58′	133°8′	84–89	1790	200	1727	20
21	Qindeli3	48°00′	133°15′	225	9523	125	9525	17
22	Fuyuan	N/A	N/A	150	9300	100	9300	17
23	Yongfa	47°00′	130°15′	N/A	5655	215	5655	20
24	Xingshu	47°04′	133°40′	60	3990	80	3990	17
25	Tongjiang	48°05′	133°15′	80	4917	120	4917	23

## References

[b1] Gorham E. Northern peatlands: role in the carbon cycle and probable responses to climatic warming. Ecol. Appl. 1, 182–195 (1991). 10.2307/1941811 27755660

[b2] Freeman C., Evans C., Monteith D., Reynolds B. & Fenner N. Export of organic carbon from peat soils. Nature 412, 785–785 (2001). 11518954 10.1038/35090628

[b3] Dise N. B. Peatland response to global change. Science 326, 810–811 (2009). 19892972 10.1126/science.1174268

[b4] Malmer N., Johansson T., Olsrud M. & Christensen T. R. Vegetation, climatic changes and net carbon sequestration in a North-Scandinavian subarctic mire over 30 years. Global Change Biol. 11, 1895–1909 (2005).

[b5] Jones M. C. & Yu Z. Rapid deglacial and early Holocene expansion of peatlands in Alaska. PNAS. 107, 7347–7352 (2010). 20368451 10.1073/pnas.0911387107 PMC2867679

[b6] Zhao Y., Yu Z. & Zhao W. Holocene vegetation and climate histories in the eastern Tibetan Plateau: controls by insolation-driven temperature or monsoon-derived precipitation changes? Quat. Sci. Rev. 30, 1173–1184 (2011).

[b7] Belyea L. R. & Malmer N. Carbon sequestration in peatland: patterns and mechanisms of response to climate change. Global Change Biol. 10, 1043–1052 (2004).

[b8] Carroll P. & Crill P. Carbon balance of a temperate poor fen. Global Biogeochem. Cycles. 11, 349–356 (1997).

[b9] Bridgham S. D., Megonigal J. P., Keller J. K., Bliss N. B. & Trettin C. The carbon balance of North American wetlands. Wetlands 26, 889–916 (2006).

[b10] Yu Z., Loisel J., Brosseau D. P., Beilman D. W. & Hunt S. J. Global peatland dynamics since the Last Glacial Maximum. Geophys. Res. Lett. 37, 1–5 (2010).

[b11] Frolking S. *et al.* Modeling northern peatland decomposition and peat accumulation. Ecosystems 4, 479–498 (2001).

[b12] Gorham E., Janssens J. A. & Glaser P. H. Rates of peat accumulation during the postglacial period in 32 sites from Alaska to Newfoundland, with special emphasis on northern Minnesota. Can. J. Earth Sci. 81, 429–438 (2003).

[b13] Ehrich D., Alsos I. G. & Brochmann C. Where did the northern peatland species survive the dry glacials: cloudberry (Rubus chamaemorus) as an example. J. Biogeogr. 35, 801–814 (2008).

[b14] Adams J. M., Faure H., Faure-Denard L., McGlade J. & Woodward F. Increases in terrestrial carbon storage from the Last Glacial Maximum to the present. Nature 348, 711–714 (1990).

[b15] An Z. S. The history and variability of the East Asian paleomonsoon climate. Quat. Sci. Rev. 19, 171–187 (2000).

[b16] Zhang. S. Q., Deng W., Yan M. H., Li X. Q. & Wang S. Z. Pollen record and forming process of the peatland in Late Holocene in the north bank of the Xingkai Lake, China. Wetland Sci. 2, 110–115 (in Chinese) (2004a).

[b17] Xia Y. M. Preliminary study on vegetaional development and climticchanges in the Sanjiang Plain in the last 12, 000 years. Sci. Geog. Sin. 8, 240–250 (in Chinese) (1988).

[b18] China Quaternary Research Committee. The Proceedings of Quaternary glacial and Quaternary Geology (in Chinese) (Geological Press, Beijing, 1988.

[b19] Zhang S. Q., Deng W. & Yan M. H. Palynological record of Dongsheng area, Baoqing since 5000a B.P. and its response to palaeoclimatic vibration. J. Jilin U. (Earth Edit.) 34, 321–325 (in Chinese) (2004b).

[b20] Li C. L., Chen B. W., Wang R. S. & Chen L. The report of ^14^C data for peat samples. Sci. Geog. Sin. 5, 86–88 (in Chinese) (1985).

[b21] Leng X. T., Li Y. Y. & Bellinge S. J. Comparison of peatification periods of holocene in the northeast of China and in Byelorussia and Analyses of its formation cause. J. NE. Norm. U. (Nat. Edit.) 1, 116–112 (in Chinese) (1997).

[b22] Yin S. C., Zhang W. C. & Chen Y. D. Chinese peat resources and exploitation (in Chinese) (Geological Press, Beijing, 1991).

[b23] Yang Y. X. & Wang S. Y. Study on mire development and paleoenvironment change since 8.0 ka B.P. in the northern part of the Sangjiang Plain. Sci. Geograph. Sin. 23, 32–38 (in Chinese) (2003).

[b24] Ma X. H., Liu X. T. & Wang R. F. China’s wetlands and agro-ecological engineering. Ecol. Eng. 2, 291–301 (1993).

[b25] Liu X. T. Wetland and its rational utilization and conservation in the Sanjiang Plain (Jilin Science Technology Press, Changchun, 1995).

[b26] Song K. S. *et al.* Land use change in Sanjiang Plain and its driving forces analysis since 1954. Acta Geog. Sin. 63, 93–104 (In Chinese) (2008).

[b27] Wang Y. J. *et al.* The Holocene Asian monsoon: links to solar changes and North Atlantic climate. Science 308, 854–857 (2005). 15879216 10.1126/science.1106296

[b28] Li S. H. & Sun J. M. Optical dating of Holocene dune sands from the Hulun Buir Desert, northeastern China. Holocene 16, 457–462 (2006).

[b29] Zhou W. *et al.* High-resolution evidence from southern China of an early Holocene optimum and a mid-Holocene dry event during the past 18,000 years. Quat. Res. 62, 39–48 (2004).

[b30] Xu H. *et al.* Holocene peatland development along the eastern margin of the Tibetan Plateau. Quat. Res. 80, 47–54 (2013).

[b31] Xiao J. L. *et al.* Holocene vegetation variation in the Daihai Lake region of north-central China: a direct indication of the Asian monsoon climatic history. Quat. Sci. Rev. 23, 1669–1679 (2004).

[b32] Sun J. M., Li S. H., Han P. & Chen Y. Y. Holocene environmental changes in the central Inner Mongolia, based on single-aliquot-quartz optical dating and multi-proxy study of dune sands. Palaeogeogr. Palaeoclimatol. Palaeoecol. 233, 51–62 (2006).

[b33] Johnson K. R. & Ingram B. L. Spatial and temporal variability in the stable isotope systematics of modern precipitation in China: implications for paleoclimate reconstructions. Earth Planet. Sci. Lett. 220, 365–377 (2004).

[b34] Zhao Y. *et al.* Peatland initiation and carbon accumulation in China over the last 50,000 years. Earth-Sci. Rev. 128, 139–146 (2014).

[b35] Song C. C., Xu X. F., Tian H. Q. & Wang Y. Y. Ecosystem–atmosphere exchange of CH_4_ and N_2_O and ecosystem respiration in wetlands in the Sanjiang Plain, Northeastern China. Global Change Biol. 15, 692–705 (2009).

[b36] Dean W. E. Determination of carbonate and organic matter in calcareous sediments and sedimentary rocks by loss on ignition; comparison with other methods. J. Sediment. Res. 44, 242–248 (1974).

[b37] Vitt D. H., Halsey L. A., Bauer I. E. & Campbell C. Spatial and temporal trends in carbon storage of peatlands of continental western Canada through the Holocene. Can. J. Earth Sci. 37, 683–693 (2000).

[b38] Stuiver M. & Reimer P. J. Extended ^14^C data base and revised CALIB 3.0 ^14^C age calibration program. Radiocarbon 35, 215–230 (2006).

